# Dual action of amitriptyline on NMDA receptors: enhancement of Ca-dependent desensitization and trapping channel block

**DOI:** 10.1038/s41598-019-56072-z

**Published:** 2019-12-19

**Authors:** Yulia D. Stepanenko, Sergei I. Boikov, Dmitry A. Sibarov, Polina A. Abushik, Nina P. Vanchakova, Daria Belinskaia, Natalia N. Shestakova, Sergei M. Antonov

**Affiliations:** 1 Sechenov Institute of Evolutionary Physiology and Biochemistry of the Russian Academy of Sciences, Torez pr. 44, Saint-Petersburg, Russia; 2 Pavlov First Saint-Petersburg State Medical University, Lev Tolstoi str. 6-8, Saint-Petersburg, Russia

**Keywords:** Ion transport, Ion channels in the nervous system, Cellular neuroscience

## Abstract

Although the tricyclic antidepressant amitriptyline (ATL) is widely used in the clinic, the mechanism underlying its high therapeutic efficacy against neuropathic pain remains unclear. NMDA receptors (NMDARs) represent a target for ATL and are involved in sensitization of neuropathic pain. Here we describe two actions of ATL on NMDARs: 1) enhancement of Ca^2+^-dependent desensitization and 2) trapping channel block. Inhibition of NMDARs by ATL was found to be dependent upon external Ca^2+^ concentration ([Ca^2+^]) in a voltage-independent manner, with an IC_50_ of 0.72 μM in 4 mM [Ca^2+^]. The ATL IC_50_ value increased exponentially with decreasing [Ca^2+^], with an *e*-fold change observed per 0.69 mM decrease in [Ca^2+^]. Loading neurons with BAPTA abolished Ca^2+^-dependent inhibition, suggesting that Ca^2+^ affects NMDARs from the cytosol. Since there is one known Ca^2+^-dependent process in gating of NMDARs, we conclude that ATL most likely promotes Ca^2+^-dependent desensitization. We also found ATL to be a trapping open-channel blocker of NMDARs with an IC_50_ of 220 µM at 0 mV. An *e*-fold change in ATL IC_50_ was observed to occur with a voltage shift of 50 mV in 0.25 mM [Ca^2+^]. Thus, we disclose here a robust dependence of ATL potency on extracellular [Ca^2+^], and demonstrate that ATL bound in the NMDAR pore can be trapped by closure of the channel.

## Introduction

More than half of patients with chronic neuropathic pain also suffer from depression^1^. Use of antidepressants as an adjuvant therapy for ameliorating chronic pain is a promising treatment strategy for patients displaying both neuropathic pain and depression^2^. However, the mechanisms underlying analgesic effects of many psychotropic drugs are currently unknown. Antidepressants represent a wide variety of compounds with diverse molecular structures, making it difficult to attribute their analgesic properties to action on specific targets. Among antidepressants with analgesic properties, amitriptyline (ATL) has long been successfully used in treatment of pain^3^.

ATL, a tricyclic antidepressant, demonstrates a broad profile of pharmacological actions. ATL effects considered to be relevant for antidepressant therapy include: 1) inhibition of monoamine uptake such as norepinephrine, dopamine, serotonin etc., 2) considerable M-cholinolytic, antihistamine and α-adrenolytic activities, and 3) block of Na^+^, K^+^ and Ca^2+^ channels [for review see^4^]. Additionally, ATL inhibits Ca^2+^ transport by Na^+^/Ca^2+^-exchanger (NCX) in synaptosomes and also demonstrates voltage- and Mg^2+^-dependent open-channel block of NMDA receptors (NMDARs)^5,6^. ATL is metabolized by the liver to nortriptyline, which further expands its wide set of molecular targets^7,8^.

It is well established that excitatory glutamatergic synaptic transmission is involved in sensitization of neuropathic pain. Nerve injury potentiates NMDAR activity at both pre- and postsynaptic sites^9,10^. In particular, injury of the dorsal root ganglion and spinal superficial dorsal horn neurons increases expression of α2δ-1^11^, a voltage-activated Ca^2+^ channel subunit that also interacts with NMDARs, promoting synaptic and surface expression of α2δ-1-NMDAR complexes and sensitization of neuropathic pain^12^. We recently described that NMDARs are upregulated by NCX in physiological conditions and that inhibition of Ca^2+^ export from neurons by the NCX inhibitor (2-[4-[(4-nitrophenyl)methoxy]phenyl]ethyl ester, methanesulfonate, KB-R7943) or lithium enhances Ca^2+^-dependent desensitization of NMDARs^13,14^. Modulation of NMDAR activity by ATL may potentially contribute to its efficacy in treating neuropathic pain. Indeed, other drugs acting directly on NMDARs (e.g., ketamine^15^ and memantine^16^) or indirectly modulating NMDAR activity (e.g., gabapentinoids, which interact with α2δ-1 and prevent synaptic expression of α2δ-1-NMDAR complexes^9^) possess analgesic potential^17^.

Despite our knowledge of the molecular targets of ATL, the high therapeutic efficacy of ATL against neuropathic pain remains a puzzle. While ATL blocks open channels of NMDARs, the precise mechanism of the blockade is still unclear. In general, compounds satisfying the “sequential” model of open-channel block^18,19^ inhibit integral NMDA-activated currents less effectively than trapping channel blockers^20–22^. Relatively large ATL concentrations (15–60 µM) are required for the NMDAR channel block^5^. However, this somewhat contradicts clinical observations that neuropathic pain relief by ATL treatment can be rapidly achieved with dosages that are lower than those required for treatment of depression^23^.

To gain insights into the mechanism of ATL action on NMDARs, we used patch-clamp electrophysiology to study the effects of ATL on whole-cell NMDAR currents in primary cultures of rat cortical neurons, which express GluN1, GluN2A, and GluN2B subunits^24^ forming diheteromeric and triheteromeric NMDARs^25,26^. We also utilize electrophysiological recordings and structural modeling of NMDARs to investigate the mechanism of ATL binding to the NMDAR channel. We disclose here a novel mode of pharmacological action of ATL, a robust dependence of ATL potency on extracellular Ca^2+^ concentration, and demonstrate that ATL causes trapping open-channel block of NMDARs, binding to a site similar to MK-801 and memantine binding sites within the NMDAR channel pore.

## Results

### Contradictions in phenomenology of NMDAR inhibition by amitriptyline

**Figure 1:**
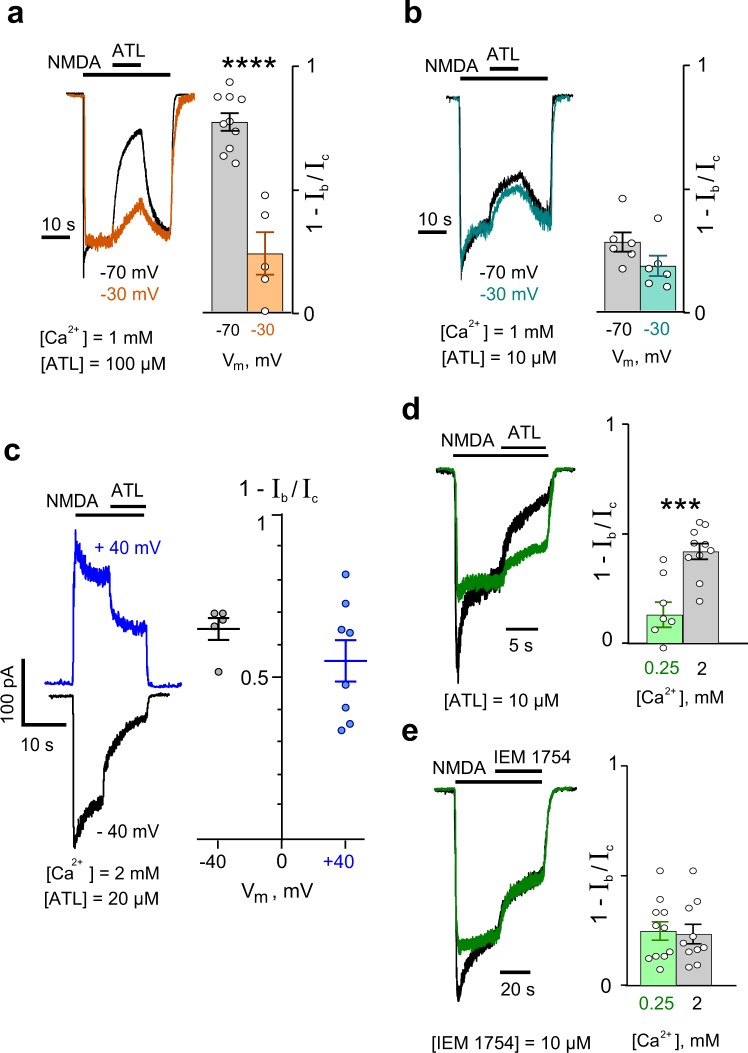
Characterization of amitriptyline (ATL) effects on NMDA-activated currents. (**a**) An overlay of normalized currents activated by 100 μM NMDA + 30 μM Gly recorded at −70 mV and −30 mV in the presence of 1 mM Ca^2+^ in the bathing solution (left). 100 μM ATL was applied at steady state. The agonist and drug applications are shown above the traces as bars. The histogram (right) depicts the fraction of blocked current (1 − I_b_/I_c_) obtained in each of experiments (circles) at −70 mV and −30 mV. Mean values ± S.E.M. are compared. ****Data are significantly different (*p* = 0.0001, Student’s two-tailed *t*-tests). (**b**) An overlay of normalized currents activated by 100 μM NMDA + 30 μM Gly recorded at −70 mV and −30 mV in the presence of 1 mM Ca^2+^ in the bathing solution (on the left). 10 μM ATL was applied at the steady state. In the histogram (right), fractions of blocked current (1 − I_b_/I_c_) obtained in each of experiments (circles) at −70 mV and −30 mV mean values ± S.E.M are depicted. Data are not significantly different (*p* = 0.12, Student’s two-tailed *t*-tests). (**c**) Currents activated by 100 μM NMDA + 30 μM Gly recorded at −40 mV and + 40 mV in the presence of 2 mM Ca^2+^ in the bathing solution (on the left). 20 μM ATL was applied as shown above the traces by bars. Plot (right) depicts fractions of blocked current (1 − I_b_/I_c_) obtained in each experiment (circles) at −40 mV and +40 mV as well as mean values ± S.E.M. Data are not significantly different (*p* = 0.19, Student’s two-tailed *t*-tests). (**d**) An overlay of currents activated by 100 μM NMDA + 30 μM Gly normalized to the steady-state amplitude recorded at −70 mV in the presence of 0.25 mM and 2 mM Ca^2+^ in the bathing solution (left). 10 μM ATL was applied as shown by bars above the traces. The histogram (right) depicts the fraction of blocked current (1 − I_b_/I_c_) obtained at −70 mV in the presence of 0.25 mM and 2 mM Ca^2+^ in the bathing solution in each of experiments (circles) as well as mean values ± S.E.M. ^***^Data are significantly different (*p* = 0.0004, Student’s two-tailed *t*-tests). (**e**) An overlay of currents activated by 100 μM NMDA + 30 μM Gly normalized to the steady-state amplitude recorded at −70 mV in the presence of 0.25 mM and 2 mM Ca^2+^ in the bathing solution (left). 10 μM IEM-1754 was applied as shown by bars above the traces. Histogram (right) depicts fractions of blocked current (1 − I_b_/I_c_) obtained at −70 mV in the presence of 0.25 mM and 2 mM Ca^2+^ in the bathing solution in each of experiments (circles) as well as mean values ± S.E.M. Data are not significantly different (*p* = 0.82, Student’s two-tailed *t*-tests).

We first examined general properties of ATL inhibition of whole-cell currents activated by NMDA in cortical neurons that suggest ATL has multiple mechanisms of action on NMDARs. 100 μM ATL, with 1 mM Ca^2+^ in the external solution, caused block of NMDAR currents that depended on membrane voltage. Fractions of blocked currents (see ‘Methods‘, ‘Analysis of membrane currents‘) were considerably larger at −70 mV than at −30 mV (Fig. 1a, Supplementary Table 1). As voltage dependence represents a key feature characterizing an open-channel blocker, the above observation suggests that ATL is an open-channel blocker of NMDARs. However, when the effect of 10 μM ATL was tested under the same conditions, voltage dependence was not observed. Fractions of blocked currents were similar both at −70 mV and at −30 mV (Fig. 1b). Furthermore, we did not find any voltage dependence in a wide membrane voltage range from −40 mV to +40 mV when NMDAR currents were inhibited by 20 μM ATL in an external solution containing 2 mM Ca^2+^ (Fig. 1c). This observation could not be easily explained within the framework of the open-channel block mechanism, unless the blocking molecule binds to a site that does not sense the plasma membrane electric field in the NMDAR external vestibule.

In the above experiments, we noticed that inhibition by lower (10 μM, 20 μM) ATL concentrations appeared to be facilitated in 2 mM Ca^2+^ (Fig. 1c) compared to 1 mM Ca^2+^ (Fig. 1b), suggesting possible involvement of extracellular Ca^2+^ in NMDAR block by ATL. We therefore examined ATL inhibition of NMDAR currents as a function of external Ca^2+^ concentration ([Ca^2+^]). Indeed, 10 μM ATL inhibited NMDAR currents in a Ca^2+^-dependent manner, with inhibition by ATL being far more pronounced in 2 mM Ca^2+^- than in 0.25 Ca^2+^- containing media (Fig. 1d).

In general, it cannot be excluded that Ca^2+^, as a permeant ion of NMDAR channels, could affect open-channel block. It is well established that the parameters of channel block of NMDARs by a blocking molecule^27^ and/or Mg^2+^^28,29^ are strongly affected by permeant ions. In addition, several binding sites for Ca^2+^ were found in the ion pathway of NMDARs^30,31^ that also likely interact with blocking molecules within the ion pore of NMDARs. A lack of information on this subject forced us to perform experiments with *N*-(tricyclo[3.3.1.13,7]dec-1-ylmethyl)-1,5-pentanediamine dihydrobromide (IEM-1754^32^), a “pure” open-channel blocker that can bind to a shallow binding site in the NMDAR channel, preventing closure of the channel, as well as a deeper binding site, where it can be trapped in the channel^19,27^. Effects of 10 μM IEM-1754 on NMDA-activated currents were examined in the presence of 2 mM Ca^2+^ and 0.25 mM Ca^2+^ in the external solution. Figure 1e demonstrates that IEM-1754 caused block of NMDA-activated currents to a similar extent in both 2 mM Ca^2+^ and 0.25 mM Ca^2+^, suggesting that parameters of general open-channel block are not affected by extracellular Ca^2+^ within this [Ca^2+^] range. This later observation raises a question about the origin of the discrepancy we observed between the effects of low (10 μM, 20 μM) and high (100 μM) ATL concentrations on NMDARs - how could Ca^2+^ be contributing to inhibition by ATL?

### Dependence of amitriptyline inhibition of NMDARs on external Ca^2+^

**Figure 2:**
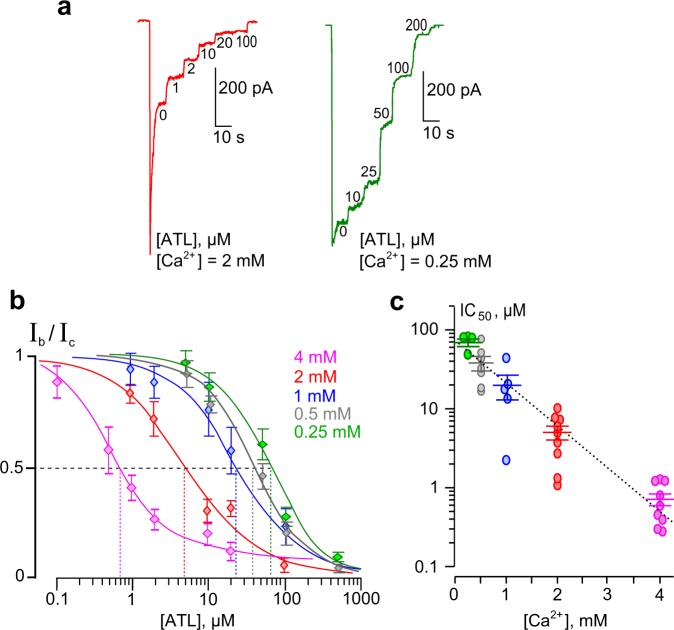
Ca^2+^-dependent inhibition of NMDAR currents by amitriptyline (ATL). (**a**) Currents activated by 100 μM NMDA + 30 μM Gly recorded at −70 mV in the presence of 0.25 mM and 2 mM Ca^2+^ in the bathing solution in the absence of ATL (0) and in the presence of rising ATL concentrations ([ATL]) indicated by numbers at corresponding level of currents (in μM). (**b**) Concentration-inhibition curves for ATL for currents activated by 100 μM NMDA + 30 μM Gly recorded at −70 mV in the presence of different [Ca^2+^]s in the bathing solution. Symbols show mean values ± S.E.M of the relative amplitudes of currents (I_b_/I_c_) in the presence (I_b_) and absence (I_c_) of different ATL concentrations ([ATL]) from 5–9 measurements. Solid lines are fits to the data with the Hill equation (Eq. 1). (**c**) Dependence of IC_50_ (a concentration that causes a half-maximal inhibition) for ATL inhibition of NMDAR currents on external [Ca^2+^] obtained from experiments illustrated in panels **a** and **b**. Data from each experiment (symbols) and mean values ± S.E.M. are shown. The IC_50_ values are 63.0 ± 9.3 μM, *h* = 1.4 ± 0.1 (n = 8); 37.6 ± 7.7, *h* = 1.5 ± 0.1 (n = 7); 21.6 ± 8.7 μM, *h* = 1.6 ± 0.5 (n = 5); 4.9 ± 1.0 μM, *h* = 1.2 ± 0.1 (n = 9); and 0.72 ± 0.12 μM, *h* = 1.5 ± 0.2 (n = 10) in the presence of 0.25 mM, 0.5 mM, 1 mM, 2 mM and 4 mM Ca^2+^ in the bathing solution, respectively. The dotted line is an approximation of the data by a single exponential function. An *e*-fold change in IC_50_ is achieved by a shift of [Ca^2+^] of 0.63 mM.

To quantify the effect of external [Ca^2+^] on inhibition by ATL, we studied concentration-inhibition relationships to estimate the concentration that causes half-maximal inhibition (IC_50_) in the presence of different [Ca^2+^]s in the bathing solution. In these experiments, increasing concentrations of ATL were sequentially applied during NMDA-activated currents at steady state (Fig. 2a). Amplitudes of currents were plotted as a function of ATL concentration and the IC_50_ values were measured by fitting the data to the Hill equation (Eq. 1). Increasing external [Ca^2+^] strongly decreased the IC_50_ value of NMDAR inhibition by ATL (Fig. 2b). The IC_50_ value measured in 4 mM Ca^2+^, 0.72 µM, increased over 100-fold in 0.25 mM Ca^2+^. The Ca^2+^-dependence of IC_50_ for the inhibition of NMDA-activated currents by ATL could be fit well with an exponential function (Fig. 2c, Supplementary Table 2). This dependence is rather sharp, with an *e*-fold change of the IC_50_ value achieved with a [Ca^2+^] shift of 0.63 mM. The single exponential approximation to the data (Fig. 2c) yielded the equation as follows: ${{{\rm{IC}}}_{50}}^{[{{\rm{Ca}}}^{2+}]}$  = 91 × exp (−[Ca^2+^]/0.63), where ${{{\rm{IC}}}_{50}}^{{[{\rm{Ca}}}^{2+}]=0}$ = 91 μM and 0.63 mM is the *e*-fold change value.

**Figure 3:**
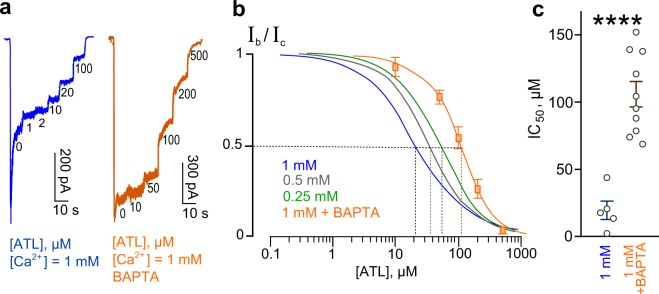
Loading neurons with BAPTA to chelate intracellular Ca^2+^ reduces ATL inhibition of NMDAR currents. (**a**) Currents activated by 100 μM NMDA + 30 μM Gly recorded at −70 mV in the presence of 1 mM Ca^2+^ in the bathing solution in the absence of ATL (0) and in the presence of rising ATL concentrations ([ATL]) indicated by numbers at corresponding level of currents (in μM). The blue trace shown was recorded from intact neuron and the orange trace - from BAPTA-loaded neuron. (**b**) Concentration-inhibition curve for ATL obtained from currents activated by 100 μM NMDA + 30 μM Gly recorded at −70 mV in the presence of 1 mM [Ca^2+^] in the bathing solution. Orange symbols represent mean values ± S.E.M of the relative amplitudes of currents (I_b_/I_c_) in the presence (I_b_) and absence (I_c_) of different ATL concentrations ([ATL]) measured from neurons loaded with BAPTA (n = 10). Solid orange line fits the data with the Hill equation (Eq. 1). The blue, gray and green curves are replotted from Fig. 2b to display the difference of results between control and BAPTA-loaded neurons. (**c**) Comparison of ATL IC_50_ values obtained in 1 mM [Ca^2+^] for control (21.6 ± 8.7 μM, n = 5) and BAPTA-loaded (105.8 ± 9.5 µM, n = 10) neurons. Data from each experiment (symbols) and mean values ± S.E.M. are shown. ^****^Data are significantly different (*p* = 0.0001, Student’s two-tailed *t*-tests).

In principle, external Ca^2+^ interactions with the Ca^2+^ binding sites found in the external vestibule of recombinant GluN1/GluN2A and GluN1/GluN2B receptors^33–35^ could contribute to the effects described above. However, Ca^2+^ influx through activated NMDAR channels could act from inside by promoting Ca^2+^-dependent desensitization. To determine whether Ca^2+^ affects NMDARs externally or internally, we performed experiments on neurons loaded with BAPTA, which chelates intracellular Ca^2+^. Figure 3a shows that in 1 mM external Ca^2+^ neurons loaded with BAPTA display far weaker desensitization of NMDARs, presumably due to ablation of Ca^2+^-dependent desensitization. In addition, a considerable increase of ATL concentration was required to inhibit currents activated by NMDA. This resulted in a shift of the concentration-inhibition relationship for ATL toward larger concentrations (Fig. 3b), so that the ATL IC_50_ in the presence of 1 mM external Ca^2+^ significantly elevated from 21.6 ± 8.7 µM (n = 5) to 105.8 ± 9.5 µM with BAPTA (n = 10, *p* = 0.0001, Student’s two-tailed *t*-tests, Fig. 3c). These data are consistent with our ideas that Ca^2+^ acts via the cytosol and that Ca^2+^-dependent desensitization of NMDARs contributes to the Ca^2+^ dependence of ATL effects on NMDARs.

We also tested whether ATL affects closed NMDARs. Regardless of [Ca^2+^] (0.25 mM or 2 mM), ATL applied prior to NMDA did not affect NMDA-activated currents. Simultaneous application of both ATL and NMDA was required for blockade. This suggests that activation of NMDARs and Ca^2+^ entry through the channels are indispensable conditions for the effects of ATL on NMDARs (Supplementary Fig. S1, Table 3).

It became clear that this pharmacological action of ATL does not fit the traditional open-channel block model and has not been described so far. Because Ca^2+^-dependent desensitization is the only known Ca^2+^-dependent process in NMDAR activation kinetics, we conclude that ATL can somehow enhance Ca^2+^-dependent NMDAR desensitization.

### Open-channel block of NMDARs by amitriptyline

**Figure 4:**
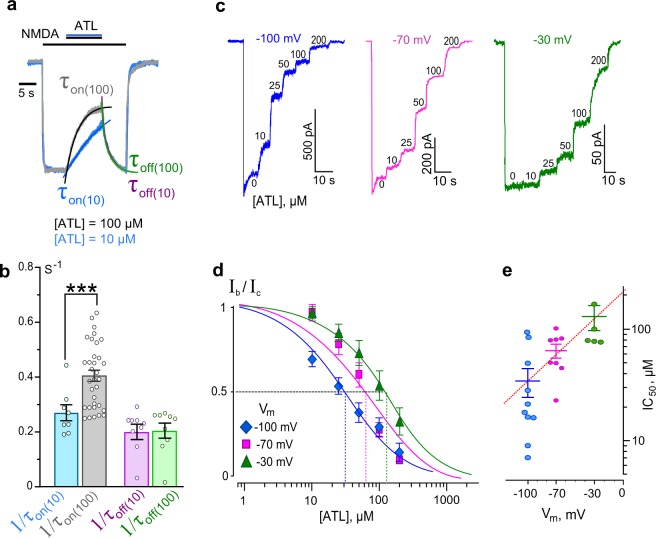
Characteristics of open-channel block of NMDARs by amitriptyline (ATL). (**a**) An overlay of normalized currents activated by 100 μM NMDA + 30 μM Gly recorded at −70 mV in the presence of 0.25 mM Ca^2+^. 10 μM or 100 μM ATL was applied at steady state. Applications of agonist and drug are shown above the traces as bars. The onset and offset of ATL block of currents were well fitted by a single exponential function (smooth lines through corresponding currents), yielding time constants for block (τ_on_) and unblock (τ_off_). (**b**) Histogram representing a comparison of rates of block onset (1/τ_on_) and offset (1/τ_off_) obtained for 10 μM and 100 μM ATL at −70 mV. Values (circles) from each experiment, illustrated in panel a, as well as mean values ± S.E.M. are compared. ^***^Data are significantly different (*p* = 0.0003, Student’s two-tailed *t*-tests). The K_d_ value for ATL, calculated using Eqs. 3, 4 and 5, is 127.5 μM. (**c**) Currents activated by 100 μM NMDA + 30 μM Gly recorded in 0.25 mM Ca^2+^ at −100 mV, −70 mV and −30 mV in the absence of ATL (0) and in the presence of rising ATL concentrations ([ATL]) indicated by numbers at corresponding level of current (in μM). (**d**) Concentration-inhibition curves for ATL for currents activated by 100 μM NMDA + 30 μM Gly recorded at −100 mV, −70 mV and −30 mV in the presence of 0.25 mM Ca^2+^. Symbols depict mean values ± S.E.M of relative amplitudes of currents (I_b_/I_c_) measured in the presence (I_b_) and absence (I_c_) of different ATL concentrations from 5–10 experiments. Solid lines are fits to the data with the Hill equation (Eq. 1). (**e**) Voltage dependence of the IC_50_ value for ATL inhibition of NMDA-activated currents. Values (circles) from each experiment and mean values ± S.E.M. are shown. Dotted line depicts fit to the data with Eq. 6, which yielded IC_50_ (0) = 220 μM and a *e*-fold change of the IC_50_ of 50 mV.

To study open-channel block of NMDARs by ATL, we performed experiments using 0.25 mM Ca^2+^-containing bathing solution to avoid complications that could be caused by Ca^2+^-dependent processes. Under these conditions, we tested whether ATL-induced block manifestations correspond well to basic predictions of open-channel block. ATL applied at steady state of NMDAR currents induced a decrease in steady-state current. The degree of block was dependent on ATL concentration (Fig. 4a). The block onset (τ_on_) and offset (τ_off_) were well fit by a single exponential function (Eq. 2). The τ_on_ value decreased with a concentration increase whereas the τ_off_ value did not depend on ATL concentration (Fig. 4b). These data allow an estimation of ATL binding parameters with NMDAR channels at −70 mV: 0.0016 ± 0.00009 s^−1^ µM^−1^ (n = 7) for the rate constant of binding (k_on_, Eq. 3), 0.204 ± 0.005 s^−1^ (n = 7) for the rate constant of dissociation (k_off_, Eq. 4) and 127.5 ± 7.8 µM (n = 7) for the apparent equilibrium dissociation constant (K_d_, Eq. 5).

An important feature of open-channel block is voltage-dependence. We therefore studied the dependence of concentration-inhibition relationships for ATL on holding potential. In 0.25 mM Ca^2+^-containing bathing solution, we sequentially applied increasing concentrations of ATL during NMDA-activated currents at steady state while holding the cell at −30 mV, −70 mV and −100 mV. To achieve a similar extent of block with depolarization, larger ATL concentrations were required (Fig. 4c), which is readily observed by comparing the concentration-inhibition curves obtained at different holding potentials (Fig. 4d). Therefore, the IC_50_ value for ATL block of NMDAR channels is voltage-dependent and could be well fit by an exponential (Fig. 4e, Supplementary Table 2). The dependence is rather weak - an *e*-fold change of the IC_50_ value could be achieved with a holding potential shift of 50 mV.

The widely accepted interpretation of the voltage-dependence of open-channel block is based on the Woodhull model^36^, which states that since a blocking molecule binding site is located within the ionic pathway, a blocker must traverse some fraction of the membrane electric field (δ) to reach its binding site. The δ value, therefore, reflects the depth of a blocker’s binding site in the voltage field (i.e. depth in the channel) and can be estimated using Eq. 6. We estimated δ and IC_50_ (0 mV), the two important parameters that characterize voltage dependence of NMDAR blockade, for ATL to be 0.51 and 220 μM, respectively, yielding the following equation: IC_50_^Vm^ = 220 × exp (V_m _• 0.021). The fact that the open-channel block of NMDARs by ATL is well measurable on macroscopic currents provides some clues for understanding whether a blocking molecule interacts with channel gating during open-channel block.

### Trapping channel block of NMDARs by amitriptyline

Chemically diverse organic compounds can bind within the ionic pore after channel opening and inhibit current flow through NMDARs. Channel blockers differ with respect to their interaction with channel gaiting and an ability to prevent channel closure. Blockers that satisfy the sequential scheme of open-channel block (a so-called “foot-in-the-door” mechanism) interact with gaiting and prevent channel closure. While at a single-channel level this manifests in the prolongation of burst durations^18,19^, at a whole-cell level, macroscopic currents exhibit “tails” upon the simultaneous removal of agonists and a blocker^37–40^. An appearance of “tail currents” also suggests that NMDAR agonists are locked in their binding sites as long as a blocking molecule has not left the ionic pore^39,40^. The sequential model, in addition, predicts that a blocker K_d_ measured from the single-channel study should be much smaller than a blocker IC_50_ estimated from macroscopic currents^41^. Because ATL did not cause tail currents, we examined whether ATL can be trapped within the ionic pore of NMDARs by channel closure. Many other NMDAR channel blockers exhibit trapping, including Mg^2+^ ^42^, MK-801^20^, phencyclidine, ketamine, amantadine, memantine^21,22^, and argiotoxin-636^43^, a spider neurotoxin that blocks ionotropic glutamate gated channels^44,45^.

**Figure 5:**
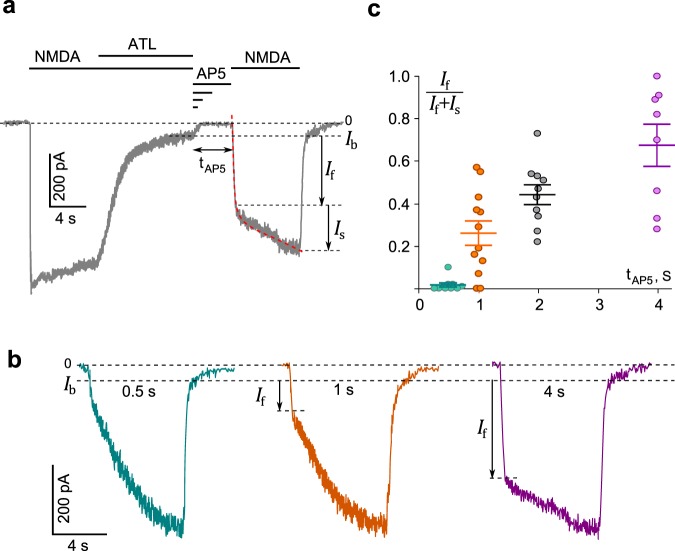
Trapping channel block of NMDARs by amitriptyline (ATL). (**a**) An example of whole-cell currents activated by 100 μM NMDA + 30 μM Gly recorded in a neuron at −70 mV in the presence of 0.25 mM Ca^2+^. When the current reached steady state, 200 μM ATL was added for 10 s, which caused a decrease of the current. After a washout of agonists and ATL of duration (t_AP5_) 0.5 s, 1 s, 2 s or 4 s with a bathing solution containing 50 μM AP5, a testing application of 100 μM NMDA + 30 μM Gly was given. Applications are shown as black bars above the trace. The rise time of the current activated by the testing application was well fitted by a double exponential function (Eq. 7, dotted line) that yielded amplitudes of fast (I_f_) and slow (I_s_) components, and their time constants. (τ_f_ and τ_s_). (**b**) Currents from a neuron activated by a testing application (100 μM NMDA + 30 μM Gly) recorded at −70 mV in 0.25 mM Ca^2+^ after washout durations of 0.5 s, 1 s and 4 s. Amplitudes of fast component (I_f_) of the rise times are indicated by arrows. (**c**) The dependence of I_f_ contribution on the amplitude of testing current measured as I_f_/(I_f_ + I_s_) on a delay duration (t_AP5_). Circles are single experimental measurements, from which mean values ± S.E.M. were calculated.

To address this point, we performed experiments in 0.25 mM Ca^2+^-containing bathing solution to minimize Ca^2+^-dependent desensitization of NMDARs using the protocol described in previous studies^20,22,43^. Application of NMDA elicited an inward current that rose rapidly to a peak and then decayed to a steady state. When 200 µM ATL was added to NMDA, the current was progressively blocked to a small residual component (I_b_, Fig. 5a). After washout of agonist and ATL with a bathing solution containing 50 µM AP5 ((2 R)-amino-5-phosphonovaleric acid, a competitive antagonist of NMDAR glutamate binding sites used to prevent random opening of NMDARs by contaminating NMDA) (t_AP5_, Fig. 5a), a test application of NMDA was applied. This process was repeated multiple times on the same neuron, with the duration of ATL and agonist washout time (t_AP5_) increasing with each run.

From the representative current trace shown on Fig. 5a, it is clear that washout of NMDA and ATL does not evoke a generation of “tail current”, suggesting that ATL is trapped inside the NMDAR channel by channel closure. The testing NMDA pulse applied after a 0.5 s washout caused a slowly rising current reflecting ATL unblock from the channels (Fig. 5b) which, if excluding a small fraction of non-blocked NMDARs (I_b_), was well fit by a single-exponential function. When washout was prolonged to 1 s, 2 s and 4 s, the rise time of currents activated by the testing NMDA pulse became biphasic, with a fast component (τ_f_) that resembled the rise time of NMDA-activated currents recorded under the control conditions (Fig. 5a) and a slow component (τ_s_) that corresponded well to ATL unblock kinetics. Fitting these data with a double-exponential function yielded the rise time constants and amplitudes (I_f_ and I_s_) for the fast and slow components. Prolongation of the washout was accompanied by a progressive increase of I_f_ and a corresponding decrease of I_s_ (Fig. 5c), while τ_f_ and τ_s_ did not depend on the washout duration (Fig. 5b). The τ_s_ values were 11 ± 3.2 s (n = 10), 13 ± 5.7 s (n = 12), 13.2 ± 4.0 s (n = 10) and 15 ± 2.9 s (n = 8) for 0.5 s, 1 s, 2 s, and 4 s delays, respectively, and did not differ significantly (Fig. 5a,b; p > 0.91 ANOVA). The increase of I_f_ with the longer washout durations could suggest that the blocking ATL molecules can somehow escape the closed channels, resulting in the fraction of unblocked channels increasing during longer washouts despite the absence of agonists and the presence of AP5 (Fig. 5a). The observation of the residual current (I_b_) in the presence of large ATL concentration (200 µM) and the relatively fast escape of ATL molecules from closed channels could presumably reflect some slow permeation of ATL and the ability to dissociate when trapped. Thus, we conclude that ATL is an NMDAR open channel blocker that displays “partial trapping”, a profile shared with other clinically relevant NMDAR channel blockers such as memantine and amantadine^22^.

### Modeling of amitriptyline binding site within NMDAR channel

Recently, it has been shown that NMDARs containing GluN2B subunits transfer the majority (about 80%) of NMDA-activated whole-cell currents in cortical neurons in primary culture^46–48^. In order to determine the structure of the ATL binding site in the ionic pore of NMDARs, we performed 3D structural modeling of a diheteromeric GluN1/GluN2B NMDAR followed by molecular docking of ATL into the NMDAR channel.

**Figure 6:**
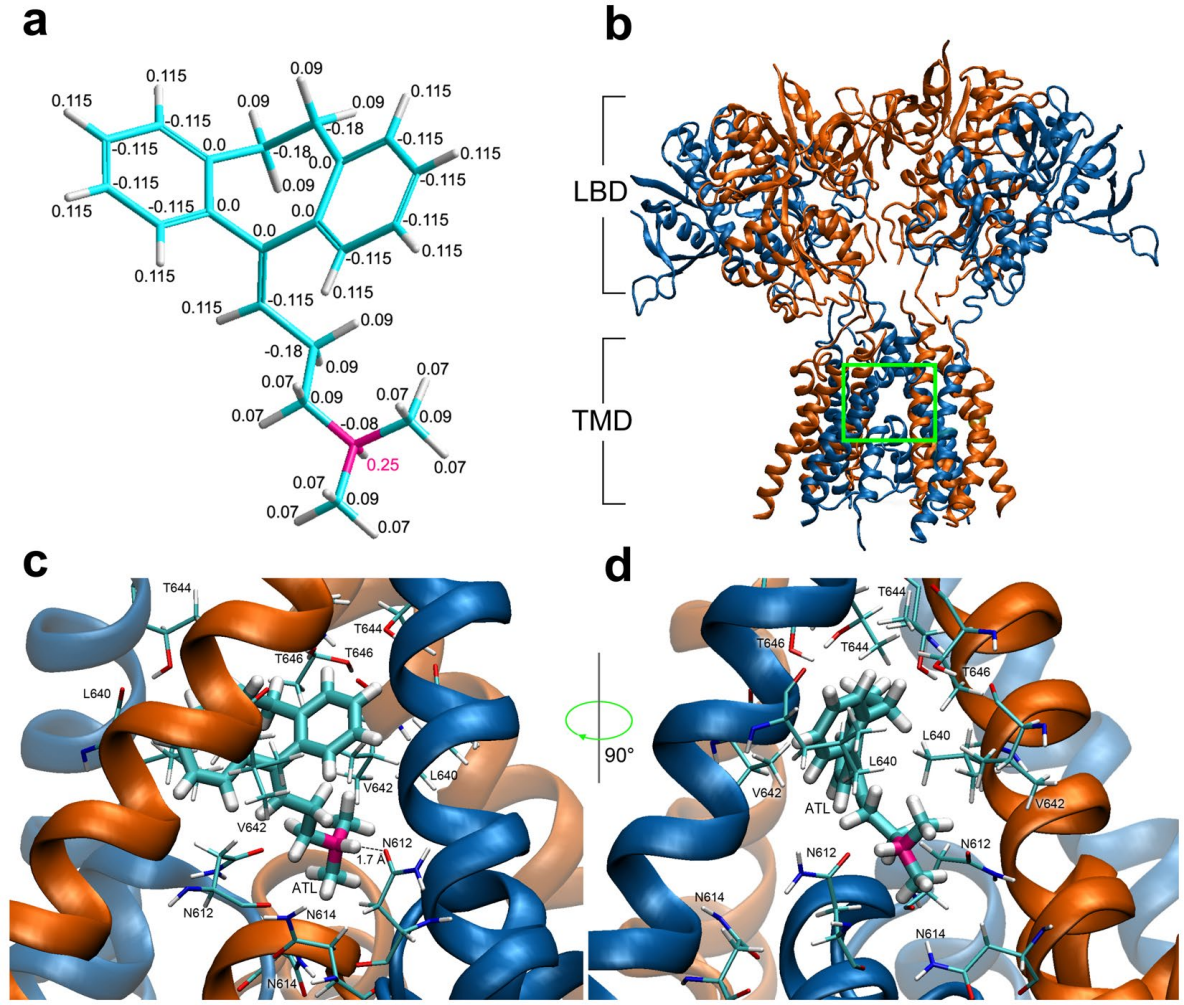
Molecular modeling of amitriptyline (ATL) interactions with the NMDAR ionic pore. (**a**) Charges of ATL atoms in the CHARMM27 force field. The carbon, hydrogen and nitrogen atoms are shown by blue, white and pink, respectively. The fractions of charge of individual atoms are indicated by numbers. Charge fraction of the positively charged hydrogen atom responsible for hydrophilic interaction with the NMDAR selectivity filter is printed in pink. (**b**) NMDA receptor lacking the amino terminal domain (pdb code 5UN1^59^). Chains А and C (GluN1 subunits) are presented in orange; chains B and D (GluN2B subunits) are presented in blue. Ligand binding domains (LBD) and transmembrane domains (TMD) are denoted. Green square indicates the region of TMD external vestibule presented at higher space resolution in panels b and c. (**c**) ATL molecule binding site in of NMDAR channel predicted by molecular docking and molecular dynamics optimization. The key amino acids are shown (presented in thin sticks): N614, V642 and T646 of Chains A and C (GluN1); N612, L640 and T644 of Chains B and D (GluN2B). The distance between the hydrogen atom of amino group of ATL and the oxygen atom of N612 (Chain D, GluN2B) is shown by a dotted line. The estimated distance between these atoms is 1.7 Ǻ. (**d**) ATL molecule binding site in the NMDAR channel rotated 90° from projection shown in (**c**).

Docking simulations predict that the ATL molecule generally exists in a single, energetically preferred binding pose. In this pose, the tricyclic group is stabilized in a V-like conformation which consists of two centralized aromatic rings, the hydrophobic part of the molecule, situated 120 degrees apart. The amine group of ATL is located distantly at the end of an aliphatic chain of 5 Å in length consisting of 3 carbon atoms (Fig. 6a). Calculations of pKa of ATL revealed that 99% of molecules exist in the protonated form at the physiological pH range (7.2–7.4) with the positive charge distributed around the molecule (Fig. 6a).

The NMDAR transmembrane domain (TMD) consists of M1–M4 helices of GluN1 and GluN2 subunits (Fig. 6b). The upper portion of the permeation pathway is a wide vestibule formed by residues in the M1, M3, and M4 helices. This vestibule is located superficially to a thin portion of the channel (the selectivity filter), which is formed by p-loops formed by the M2 segments of the transmembrane domains. Docking simulations predicted an energetically favorable binding pose for ATL in which the V-like group associates with the upper, wide portion of the vestibule while the amine group associates with the selectivity filter. The docked binding pose was found to be highly consistent with the NMDAR-ATL complex obtained by molecular dynamics (MD) simulations (Fig. 6c,d). MD simulations demonstrated that the hydrogen atom of the amine of ATL interacts with the oxygen atom of pore-loop asparagine residue N612 of one of two GluN2B subunits at a distance of 1.7 Å (Fig. 6c), a distance consistent with mutual atomic locations that allow the formation of a hydrogen bond. The V-like group of the ATL molecule is stabilized within the channel by hydrophobic interactions with surrounding and uncharged aliphatic amino acids in the M3 helices of all four subunits. M3 residues found to interact with the V-like group are GluN1(V642),GluN1(T646), GluN2B(L640), and GluN2B(T644).

## Discussion

To clarify the mechanism of ATL effects on NMDARs, here we studied the dependence of ATL inhibition of NMDARs on membrane voltage and extracellular [Ca^2+^]. We observed a peculiarity in the discrepancy between the effects of low (10 μM) and high (100 μM) ATL concentrations. Notably, 10 μM ATL in the presence of 1 mM Ca^2+^ in the bathing solution inhibited NMDAR currents in a voltage-independent manner, and changes of extracellular [Ca^2+^] in any direction considerably altered magnitude of inhibition by ATL. In contrast, blockade of NMDAR currents by 100 μM ATL was voltage-dependent, which pointed toward ATL acting as an open-channel blocker, in agreement with a previous study^5^. To address whether these contradictory observations could be explained in terms of the open-channel block, we tested how external [Ca^2+^] could influence NMDAR inhibition by IEM-1754, a “pure” open-channel blocker without any other known mechanism of action^19^ on NMDARs. Blockade of NMDAR currents by IEM-1754 was resistant to a 4-fold increase of [Ca^2+^] in the external solution, suggesting a lack of Ca^2+^ effects on blockade. Similarly, the NMDAR channel blocker ketamine^21^ shows no sensitivity to changes in external [Ca^2+^] while memantine^22^ slows the rate of recovery from desensitization of GluN1/GluN2A NMDARs (but not GluN1/GluN2B receptors) in a Ca^2+^-dependent manner, suggesting that memantine stabilizes a Ca^2+^-dependent desensitized state of GluN1/GluN2A receptors^49^. In general, NMDAR open-channel block by a variety of structurally different compounds is not affected by external [Ca^2+^] to the extent displayed by ATL. Considering the strong dependence of the IC_50_ value of ATL on external [Ca^2+^], with a 1 mM increase of external [Ca^2+^] inducing a roughly 5-fold decrease of the IC_50_ for ATL inhibition of NMDAR currents, we conclude that Ca^2+^-dependent inhibition of NMDARs by ATL represents a mode of action that differs from canonical open-channel block. Since Ca^2+^-dependent desensitization is currently the only known Ca^2+^-dependent characteristic of NMDAR kinetics, we suggest that ATL promotes the Ca^2+^-dependent desensitization of NMDARs and this particular mechanism of action dominates at low ATL concentrations.

### Ca^2+^-dependent desensitization of NMDARs

The mechanism by which ATL influences Ca^2+^-dependent desensitization of NMDARs is still unclear. Ca^2+^-dependent desensitization of NMDARs is trigged by Ca^2+^ entry to the cytosol during NMDAR activation, in binding of four Ca^2+^ ions to calmodulin, and subsequent binding of Ca^2+^-bound calmodulin to the C0 motif of the GluN1 subunit intracellular C-terminal domain^50^. There are a number of potential explanations of how ATL could influence Ca^2+^-dependent desensitization. For instance, we cannot exclude the possibility that NMDARs may have additional allosteric ATL binding sites beyond the binding site in the channel that could allow ATL to stabilize NMDARs in the Ca^2+^-dependent desensitized state. In addition, interactions with the Ca^2+^ binding sites found recently in the external vestibule of recombinant NMDARs^33–35^ could contribute to our observations. However, the lack of ATL effects on closed NMDARs argues against these assumptions (Supplementary Fig. S1). Alternatively, ATL could change the local Ca^2+^ concentration around NMDAR GluN1 C-terminal domains or inhibit Ca^2+^ export from neurons by acting on NCX, as shown in synaptosomes^6^. Inhibition of NCXs promotes Ca^2+^-dependent desensitization of NMDARs due to the tight molecular localization of NMDARs and NCXs in lipid rafts^13,14^, [for review see^51^. Besides, the presence of 10 mM EGTA in the pipette solution to control bulk intracellular Ca^2+^ NMDAR currents, recorded in our experiments, exhibited pronounced Ca^2+^-dependent desensitization. This is consistent to the previous observation, that even 40 mM EGTA could not affect Ca^2+^-dependent desensitization of NMDARs^52^ because this chelator binds Ca^2+^ much slower than calmodulin. By loading neurons with BAPTA, we were able to abolish the Ca^2+^-dependent component of ATL action in the presence of 1 mM Ca^2+^, suggesting that Ca^2+^ influx through NMDARs is required for augmentation of ATL block. Whereas this interpretation is consistent with potential action on NCXs, further investigation is required to gain insight into how Ca^2+^ modulates NMDAR inhibition by ATL.

### Trapping channel block of NMDARs

To obtain “proper” parameters of open-channel block by ATL, we performed experiments in 0.25 mM Ca^2+^-containing external solution. In general, the characteristics of the blockade were: block onset (τ_on_) depended on ATL concentration; recovery from block (τ_off_) did not depend on ATL concentration; ATL IC_50_ depended exponentially on membrane voltage that yielded IC_50_(0 mV) was 220 μM (and an *e*-fold change of the IC_50_ per 50 mV). These characteristics are consistent with open-channel block. The Woodhull model^36^ states that the voltage dependence of a blocker reflects a fraction of the membrane electric field that a blocking molecule must traverse to reach its binding site (δ) within the ionic pore. For ATL, δ equals 0.51, which is slightly smaller than values for ketamine (δ = 0.55^53^,), memantine (δ = 0.65–0.91) by different estimates^22,54–57^ and amantadine (δ = 0.72^22^,). This discrepancy could be due to the distributed charge density of the ATL molecule, a feature not shared by memantine or amantadine, or possible contribution of the voltage-independent inhibition to this estimate. An important feature of ATL block is that the NMDAR channel can close with ATL still bound, trapping the ATL molecule inside the channel. However, when agonists and ATL were simultaneously removed from the external solution we observed that the fraction of unblocked channels in a testing agonist application increased with longer washouts durations. This strongly suggests that ATL escaped from many of the blocked channels in the absence of agonists. This suggests that, like memantine and amantadine^22^, ATL exhibits “partial trapping”, open-channel block of NMDARs (Supplementary Fig. S2).

### Structural modeling of amitriptyline binding site in ionic pore

The crystal structure of diheteromeric GluN1/GluN2B NMDAR accompanied with molecular dynamics simulations revealed that MK-801 and memantine bind within the vestibule of the ion channel, promote closure of the ion channel gate, and lodge between the M3-helix-bundle crossing and the M2-pore loops. The apex of the reentrant M2 is referred to as the Q/R/N site and is formed by asparagines N614 on GluN1 and N612 on GluN2B. These aspargines, together with the N + 1 site asparagines, N613 (GluN2B), are required for channel block by MK-801^58,59^. Both MK-801^59^ and memantine^57^ bind in the same channel vestibule with the positively charged amino group facing the Q/R/N sites. *In silico* modeling of ATL binding site within the GluN1/GluN2B ion channel demonstrated that the hydrophilic amine of ATL interacts with the pore-loop asparagine residue N612 of one of two GluN2B subunits. ATL is a tertiary amine and its amino group contains one hydrogen atom that can form one hydrogen bond at a time. The N-site asparagines N612 on GluN2B subunit also contribute to channel block by MK-801 and memantine^58,59^. In contrast, the secondary amine MK-801 and the primary amine memantine being protonated at physiological pH, have two hydrogen atoms covalently bound to their amino group. These hydrogens form stable hydrogen bonds with the two pore-loop asparagine residues N614 (GluN1) and N612 (GluN2B). The V-like hydrophobic part of the ATL molecule is stabilized by aliphatic amino acids V642 and T646 on M3 helixes of both GluN1 subunits. The V642 residue also contributes to stabilize the MK-801 molecule inside of the NMDAR channel^59^ interacting with its aromatic V-like group^60^. It is worth noting that MK-801 in animal models is effective against neuropathic pain, but it is not used in the clinic because of numerous side effects^21,61–63^.

Thus, our molecular docking simulations exhibited that ATL associates with a site in the channel vestibule with the positively charged amino group facing the Q/R/N site, a binding pose similar to both MK-801^58^ and memantine^56,63^. These molecules sharing an overlapping binding site is consistent with their experimentally verified similar action of trapping open-channel block of NMDARs.

**Figure 7:**
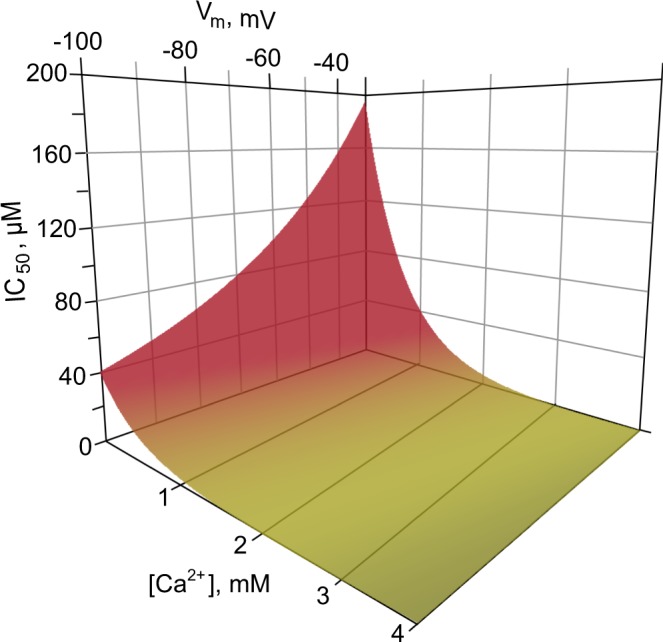
Predicted ATL IC_50_ values for NMDAR inhibition as a function of membrane voltage (V_m_) and external [Ca^2+^]. The ATL IC_50_ was calculated as ${{{\rm{IC}}}_{50}}^{{\rm{Vm}},[{{\rm{Ca}}}^{2+}]}$ = IC_50_^Vm^ × ${{{\rm{IC}}}_{50}}^{[{{\rm{Ca}}}^{2+}]}$/${{{\rm{IC}}}_{50}}^{[{{\rm{Ca}}}^{2+}]=0.25}$, where ${{{\rm{IC}}}_{50}}^{[{{\rm{Ca}}}^{2+}]=0.25}$ equals 64 µM. The final form of the equation is: ${{{\rm{IC}}}_{50}}^{{\rm{Vm}},[{{\rm{Ca}}}^{2+}]}$ = 317 × exp (V_m_ × 0.018 − [Ca^2+^]/0.69). Green color corresponds to the region of ATL therapeutic concentrations in the blood serum.

In conclusion, here we have described two mechanisms of ATL action on NMDARs. ATL is widely used in medical practice to treat a number of mental disorders and performs as a reliable drug to treat neuropathic pain. It is well established that the therapeutic window for ATL concentrations in the blood plasma is within the range of 0.3–1 µM, but in brain tissue can reach 10 µM^64^. Whereas ATL shows voltage-dependent open-channel block of NMDARs at concentrations >70 µM, at lower concentrations and in physiological levels of extracellular [Ca^2+^] (2–4 mM), ATL seems to enhance Ca^2+^-dependent desensitization of NMDARs, displaying much more potent IC_50_ values ranging from 0.7–7 µM. This concentration range is within the therapeutic window for treatment of both depression as well as neuropathic pain. Considering that open-channel block and Ca^2+^-dependent inhibition of NMDARs by ATL represent two independent processes, the experimentally measured NMDAR block is determined by additive contributions of both mechanisms. Within the physiological membrane voltages and extracellular [Ca^2+^]s, the experimentally measured value (${{{\rm{IC}}}_{50}}^{{\rm{Vm}},[{{\rm{Ca}}}^{2+}]}$) is predicted by 3-dimensional distribution (Fig. 7), calculated using equations for IC_50_^Vm^ and ${{{\rm{IC}}}_{50}}^{[{{\rm{Ca}}}^{2+}]}$ (see Results) as ${{{\rm{IC}}}_{50}}^{{\rm{Vm}},[{{\rm{Ca}}}^{2+}]}$ = IC_50_^Vm^ × ${{{\rm{IC}}}_{50}}^{[{{\rm{Ca}}}^{2+}]}$/${{{\rm{IC}}}_{50}}^{[{{\rm{Ca}}}^{2+}]}$^= 0.25 mM^, which could be presented in the final form: ${{{\rm{IC}}}_{50}}^{{\rm{Vm}},[{{\rm{Ca}}}^{2+}]}$ = 317 × exp (V_m_ × *a* − [Ca^2+^]/*b*). The *a* value, which is zδF/RT, and the *b* value estimated from experiments were 0.021 and 0.63, respectively. When these parameters were optimized by fitting all bunch of the data (Supplimentary Table 2) with this equation, the best coincidence with the experimental data was achieved for *a* = 0.018 and *b* = 0.69 (Supplementary Table 4). This correction adjusted the depth of the ATL binding site in the channel to δ = 0.75, which became similar to memantine^22,54–57^ and amantadine^22^. The 3-dimensional distribution drawn from this equation illustrates that an enhancement of Ca^2+^-dependent desensitization of NMDARs represents a mechanism of ATL action that predominantly contributes to its therapeutic usage against neuropathic pain (Supplementary Fig. S3) sensitized by a hyperfunction of NMDARs^9,10^. It is widely accepted, in addition, that calmodulin- and Ca^2+^-dependent desensitization represents a common mechanism for the receptor and channel modulation and was described for NMDARs, TRPV-channels, L-type voltage-gated channels, SK-channels, adenosine receptors and many others [for review see^51,65^]. Because ATL may promote the Ca^2+^-dependent desensitization of NMDARs, we cannot exclude that similar ATL effects on the receptors and channels mentioned above could also occur. Regardless, this mode of ATL action requires more detailed investigations.

## Methods

### Primary culture of cortical neurons

All manipulations on animals were performed in accordance with the guide of the Federation for Laboratory Animal Science Associations and were approved by the Animal Care and Use Committees of Sechenov Institute. Briefly, 16 days pregnant Wistar rats (14 female rats used in this study, supplied by the Sechenov Institute Animal Facility) were placed in a plastic box connected to a CO_2_ tank by a tube and then sacrificed by 1 min CO_2_ inhalation. Fetuses were removed and then primary cultures of rat cortical neurons were prepared using conventional procedures as described earlier^27,66^. Neurons were grown in Neurobasal culture medium supplemented with B-27 (Gibco-Invitrogen, UK) on glass coverslips coated with poly-D-lysine and were used for experiments after 10–15 days in culture^66,67^.

### Patch clamp recordings

Whole-cell currents were recorded from cultured rat cortical neurons using a MultiClamp 700B patch-clamp amplifier with Digidata 1440 A controlled by pClamp v10.2 software (Molecular Devices). Recordings were 8-order low-pass filtered at 200 Hz and the acquisition rate was 20000 samples per second. Solution exchange was performed by means of a fast solution application system as described earlier^13^. Unless otherwise specified, the external bathing solution contained (in mM): 140 NaCl; 2.8 KCl; 1.0 CaCl_2_; 10 HEPES, at pH 7.2–7.4, osmolarity 310 mOsm. The intrapipette solution contained (in mM): 120 CsF, 10 CsCl, 10 EGTA, and 10 HEPES, osmolarity 300 mOsm^14^, with pH adjusted to 7.4 with CsOH. Patch pipettes of 2–4 MΩ were pulled from Sutter BF150-89-10 borosilicate capillaries. Experiments were performed at 23–25 °C. Under control conditions, neurons were voltage clamped at −70 mV. Data are reported without corrections for liquid junction potential, which was measured as −11 mV. To activate NMDARs, 100 µM NMDA and 30 µM glycine were co-applied. Some experiments were performed on neurons loaded with BAPTA. To load BAPTA, cortical neuron cultures were incubated in bathing solution containing 10 µM BAPTA-AM for 2 hours.

### Drugs

Compounds were acquired from Sigma-Aldrich, St. Louis, MO, USA. The adamantane derivative IEM-1754 [*N*-(Tricyclo[3.3.1.13,7]dec-1-ylmethyl)-1,5-pentanediamine dihydrobromide] was synthesized by Dr. V. E. Gmiro at the Institute of Experimental Medicine, Russian Academy of Medical Sciences, St. Petersburg.

### Analysis of membrane currents

Whole-cell membrane currents activated by NMDA were measured at steady state. The fraction of blocked current was calculated as 1 − I_b_/I_c_, where I_c_ is the amplitude of current activated by agonists and I_b_ is the amplitude of current activated by agonists in the presence of blocker.

To determine amitriptyline (ATL) blocking potency, NMDA elicited currents were measured in the absence and presence of different ATL concentrations ([B]). Amplitudes of currents measured in the presence of blocker were normalized to maximal current response in control (I_c_). The IC_50_, a concentration of ATL causing 50% inhibition, and the Hill coefficient (*h*) were estimated by fitting concentration-inhibition curves with the Hill equation:

1 $${{\rm{I}}}_{{\rm{b}}}/{{\rm{I}}}_{{\rm{c}}}=1/(1+{[{\rm{B}}]}^{h}/{{\rm{IC}}}_{50}^{h}),$$

NMDAR current relaxations during block or unblock by ATL were fit with the single exponential equation:

2 $${\rm{I}}=({{\rm{I}}}_{{\rm{\max }}}-{{\rm{I}}}_{{\rm{\min }}})\cdot \,\exp \,(-{\rm{t}}/\tau )+{{\rm{I}}}_{{\rm{\min }}}$$

where I_max_ and I_min_ are maximal and minimal amplitudes of currents during relaxations and τ represents the time constant of the exponential component during block onset (τ_on_) or offset (unblock, τ_off_). From these measurements, an estimation of rate constants of block (k_on_) and unblock (k_off_) was estimated. k_on_ was obtained from τ_on_ measured at 10 μM and 100 μM ATL (C_1_ and C_2_ respectively) using the equation:

3 $${{\rm{k}}}_{{\rm{on}}}=(1/{{\rm{\tau }}}_{{\rm{onC}}2}\mbox{--}1/{\tau }_{{\rm{onC}}1})/({{\rm{C}}}_{2}\mbox{--}{{\rm{C}}}_{1})$$

The k_off_ the reciprocal to measured τ_off_:

4 $${{\rm{k}}}_{{\rm{off}}}=1/{{\rm{\tau }}}_{{\rm{off}}}$$

The equilibrium dissociation constant (K_d_) for ATL was calculated as follows:

5 $${{\rm{K}}}_{{\rm{d}}}={{\rm{k}}}_{{\rm{off}}}/{{\rm{k}}}_{{\rm{on}}}$$

To obtain the voltage dependence of NMDAR block by ATL, its concentration-response curves were generated at three membrane holding potentials V_m_ = −100, −70 and −30 mV. The obtained values were fitted with the Woodhull equation^36^:

6 $${{\rm{IC}}}_{50}({{\rm{V}}}_{{\rm{m}}})={{\rm{IC}}}_{50}(0\,{\rm{mV}})\,\cdot \,\exp \,({{\rm{V}}}_{{\rm{m}}}{\rm{z}}{\rm{\delta }}{\rm{F}}/{\rm{RT}})$$

where IC_50_(0 mV) and δ were set as free parameters. IC_50_(0 mV) is the half-maximal inhibition concentration at V_m_ = 0 mV, δ is the fraction of the membrane voltage field exerting force on ATL at its binding site, z = 1 is an ATL molecule electric charge. For Faraday constant, gas constant and room temperature of 25 °C the RT/F ≈ 25.7 mV.

The rise time of currents activated by the testing NMDA + Gly application in trapping channel block experiments was fit in ClampFit (pClamp, Axon Instruments) using the double exponential function:

7 $${\rm{I}}={{\rm{I}}}_{1}\,\cdot \,\exp \,(-\,{\rm{t}}/{{\rm{\tau }}}_{1})+{{\rm{I}}}_{2}\,\cdot \,\exp \,(-\,{\rm{t}}/{{\rm{\tau }}}_{2})-{\rm{C}}$$

where I_1_ and I_2_ are amplitudes of the exponential and τ_1_ and τ_2_ are time constants of the exponential and C equals I_1_ + I_2_ + I_b_.

### Statistical analysis

Data are presented as representative measurements as well as mean values ± standard error of the mean (S.E.M.). Sample number (n) refers to the number of recorded cells. Groups were compared using ANOVA with Bonferroni correction and Student’s two-tailed *t*-test. Statistical significance is reported in the figures according to the following symbols *** and ****, which indicate *p* values below (<) 0.001 and 0.0001, respectively. Curve fitting was performed using OriginPro software (OriginLab Corp.).

### Preparation of 3D-models

The three-dimensional model of ATL was generated from pdb entry 3APV^68^. Acid dissociation constants (pKa) of the compound and the percentage of different protonation states at neutral pH were calculated by MarvinView program 17.3.13.0 (ChemAxon Ltd., https://www.chemaxon.com/products/marvin/marvinview). There are several three-dimensional models of NMDAR in Protein Data Bank^69^. For our molecular modeling experiments, we used a model of NMDAR of African frog *Xenopus laevis*, with a molecule of dizocilpine (MK-801) bound inside the receptor channel. The code of the structure is 5UN1^70^.

The chosen receptor model 5UN1 consists of two molecules of NMDAR in the crystalized unit containing the following polypeptide chains: chains A,C,E,G (GluN1 subunits) and chains B,D,F,H (GluN2B subunits). Subunits were expressed lacking the amino-terminal domains (∆ATD receptor) to facilitate structural analysis and crystallized with glycine, glutamate and MK-801. Its three-dimensional structure was determined by diffraction method with the resolution of 3.5 Å^59^.

For the molecular modeling experiments, we used one of the two NMDAR molecules of 5UN1 structure: chains A, C (GluN1) and B, D (GluN2B). Only the moieties of the receptor subunits that form the channel pore were used for simulation: amino acids 600–653 of GluN1 subunits and amino acids 598–653 of GluN2B subunits. Hereinafter, the numbering corresponds to the primary sequence of the receptor according to UniProt database:^70^ code for A0A1L8F5J9 GluN1 and code A7XY94 for GluN2B subunits. We removed all other amino acids and ligands from the structure. Missing atoms were added using the VMD software^71^ and the structure was optimized by energy minimization using NAMD software^72^.

### Molecular docking procedure

Molecular docking of ATL into the NMDAR channel was performed using the Autodock Vina 1.1.2 software package^73^. The coordinates of the nitrogen atom of MK-801 in 5UN1 was used as the center of the Autodock search area. The search area size was set to 15 × 15 × 40 Å^3^, which covers the entire channel. The “exhaustiveness” parameter, which represents the amount of computational effort, was set to 24. The parameter “energy range”, characterizing the maximum scatter of energy values of conformations in the output file, was set to 3 kcal/mol. The number of the most optimal conformations in the output file (num_modes) was set to 10. The conformation with the best value of the binding energy was chosen for further optimization by molecular dynamics (MD) simulations.

### Molecular dynamics simulations

The structure obtained by molecular docking was optimized with a 2 ns MD simulation with the help of NAMD software^72^ using CHARMM27 force filed^73^. Partial charges of atoms in the ATL molecule used in the CHARMM force field are presented in Fig. 6a. The complex was placed into a 1-palmitoyl-2-oleoyl-sn-glycero- 3-phosphatidylcholine (POPC) lipid bilayer with the help of the “Membrane” plug-in included in the VMD software package. The system was then solvated via the “Solvate” plug-in of the VMD suite^74^. 0.15 mol/L of NaCl was added to make the system neutral. The resulting structure had a total of 103 POPC lipid molecules, 20 sodium ions, 21 chloride ions and 7153 water molecules.

The MD simulation was preceded by a three-stage relaxation of the system. At the first stage, all atoms were fixed except the tails of the POPC molecules, which were relaxed by 1000 steps of conjugate gradient energy minimization followed by 500 ps of equilibration. At the second stage, NMDAR and ATL molecules were constrained, the energy of the system was minimized, and the system was equilibrated for 500 ps. At the final stage of the relaxation procedure, all molecules were released, and the system was equilibrated for 500 ps. Following the preparation stages, a 2 ns MD simulation was run with a time step of 2 fs. Temperature (310 K) and pressure (1 bar) were kept constant using a Langevin thermostat and piston methods. The particle-mesh Ewald method (PME)^75^ with nonbonded cutoff of 12 Å was applied to compute long-range electrostatic interactions. Periodic boundary conditions were used.

## Supplementary information

supplementary information

## Data Availability

The dataset generated and analyzed in this study is available upon request to the corresponding author.

## Code availability

All code used in this study is available upon request to the corresponding author.
