# Supplementary material for: Dual action of amitriptyline on NMDA receptors: enhancement of Ca-dependent desensitization and trapping channel block
Source: Sci Rep. 2019 Dec 19;9:19454. doi: 10.1038/s41598-019-56072-z (PMC6923474; doi:10.1038/s41598-019-56072-z)
Supplement: Supplementary file 1 — supplementary information [file 41598_2019_56072_MOESM1_ESM.pdf]

Dual action of amitriptyline on NMDA receptors: enhancement of Ca-dependent desensitization and trapping channel block

Yulia D. Stepanenko[1], Sergei I. Boikov[1], Dmitry A. Sibarov[1], Polina A. Abushik[1], Nina P. Vanchakova[2], Daria Belinskaia[1], Natalia N. Shestakova[1] and Sergei M. Antonov[1*]

Supplementary Information

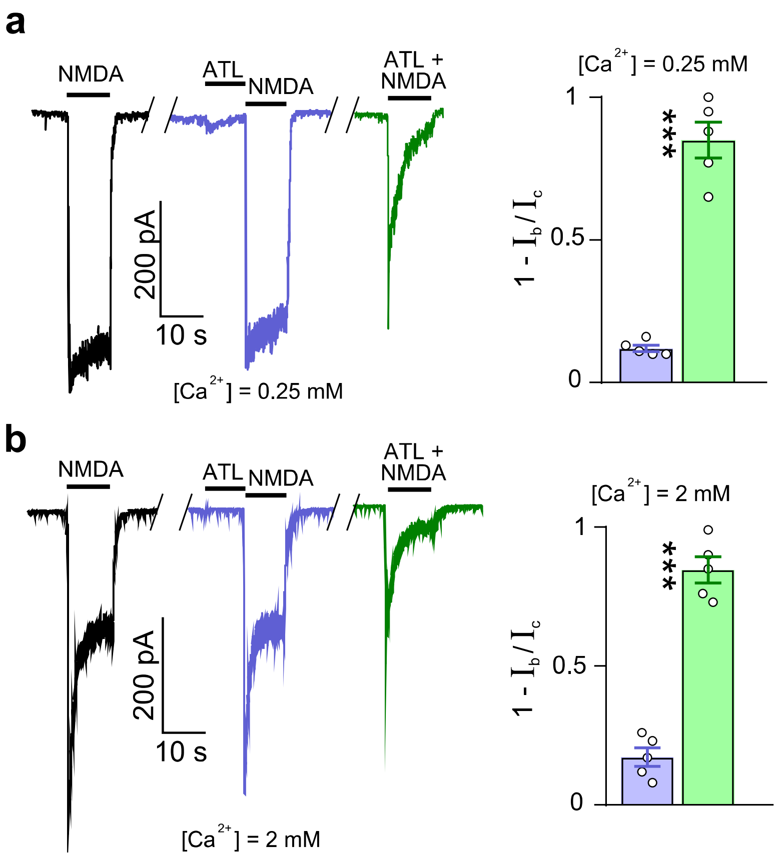

Figure S1. The lack of closed channel block of NMDARs by 100 µM amitriptyline (ATL). Examples of currents activated by agonists (100 µM NMDA + 30 µM Gly) recorded at – 70 mV obtained in the same neuron without (black), after the pre-application with ATL (blue) and on simultaneous applications of agonists and ATL (green) in the presence of 0.25 mM Ca^{2+} **(a)** or 2 mM Ca^{2+} **(b)** in the bathing solution. Agonists and ATL applications are indicated above the traces by bars. Histograms (to the right of traces) exhibit fractions of blocked current ($1-I_b/I_c$) obtained in each of experiments (circles) as well as mean values ± S.E.M. The steady state amplitude of currents activated by agonists (I_c) and the steady-state amplitudes of currents obtained following pre-application of ATL and caused by combined applications of agonists and ATL (I_b) were measured. *** - data are significantly different (for both histograms $p = 0.0004$, n = 5, Student's two-tailed *t*-tests).

Pre-application of ATL did not significantly affect currents activated by NMDA in either 0.25 mM or 2 mM Ca^{2+}, suggesting that ATL is not able to act on closed NMDARs. Combined applications of agonists and ATL, however, induced typical block of currents. This allows us to conclude that the ATL block of NMDARs requires NMDAR activation.

Table 1.

Fractions of ATL inhibition of NMDAR currents ($1-I_b/I_c$), which were pooled to plot figure 1 d.

	ATL 10 μM		ATL 100 μM	
V_m	$[Ca^{2+}]$ 0.25 mM	$[Ca^{2+}]$ 2 mM	$[Ca^{2+}]$ 0.25 mM	$[Ca^{2+}]$ 2 mM
-70 mV	0.13 ± 0.06 (*n* = 8)	0.42 ± 0.04 (*n* = 10)	0.79 ± 0.05 (*n* = 8)	0.79 ± 0.03 (*n* = 13)
-30 mV	0.12 ± 0.05 (*n* = 10)	0.30 ± 0.05 (*n* = 5)	0.40 ± 0.07 (*n* = 11)	0.35 ± 0.04 (*n* = 11)

For note, the values obtained at -30 mV in the presence of 2 mM $[Ca^{2+}]$ in 10 μM and 100 μM ATL are not significantly different (p = 0.99, ANOVA with Bonferroni post-*hoc* test). The IC_{50}^{Vm} value at -30 mV is 129 μM which is larger, than [ATL]s used. This suggests a weak contribution of the open-channel block as compared to the contribution of Ca^{2+}-dependent process for which the $IC_{50}^{[Ca2+]}$ value at 2 mM Ca^{2+} is 4.9 μM. This value is twice as lower as 10 μM and much lower, than 100 μM ATL. Therefore, the effect of 10 μM ATL under these particular conditions is already close to a saturation and an increase of [ATL] could add a little to the ATL effect.

Table 2.

The IC_{50} values pooled to plot figure 2 and figure 3e.

V_m	$[Ca^{2+}]$, mM				
	0.25	0.5	1	2	4
- 100 mV	34.2 ± 9.8 (*n* = 10)		15.3 ± 4.0 (*n* = 4)		
- 70 mV	63.0 ± 9.3 (*n* = 8)	37.6 ± 7.7 (*n* = 7)	21.6 ± 8.7 (*n* = 5)	4.9 ± 1.0 (*n* = 9)	0.72 ± 0.12 (*n* = 10)
- 30 mV	128.9 ± 32.6 (*n* = 6)		41.1 ± 6.0 (*n* = 4)		

Table 3.

Fractions of ATL inhibition of NMDAR currents ($1-I_b/I_c$), which were pooled to plot figure S1.

$[Ca^{2+}]$	Pre-application of ATL	Co-application of agonists and ATL
0.25 mM	0.12 ± 0.01 (*n* = 5)	0.85 ± 0.06 (*n* = 5)
2 mM	0.17 ± 0.03 (*n* = 5)	0.85 ± 0.05 (*n* = 5)

Table 4.

The IC_{50} values predicted by equation used to plot the 3-dimensional distribution on Fig. 7.

V_m	$[Ca^{2+}]$, mM					
	0	0.25	0.5	1	2	4
-100 mV	52.4	36.5	25.4	12.3	2.9	0.2
-70 mV	89.9	62.6	43.6	21.1	5.0	0.3
-30 mV	184.7	128.6	89.5	43.4	10.2	0.6

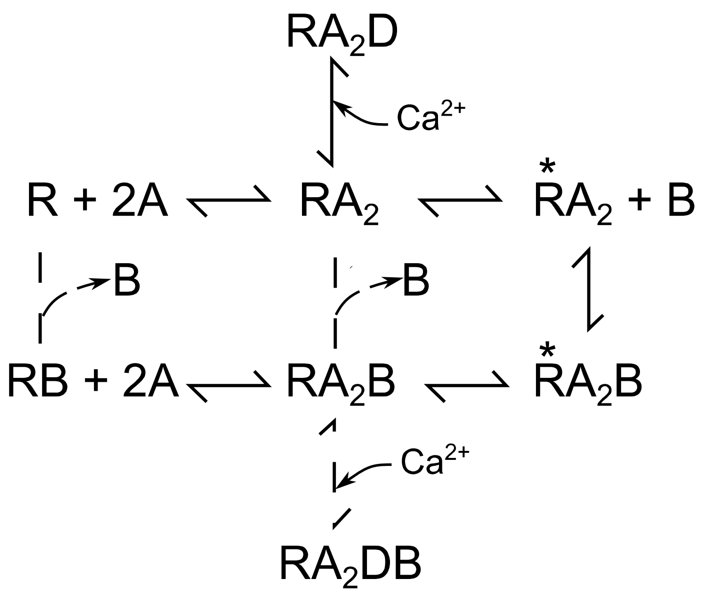

Figure S2. The kinetic model of trapping block of NMDARs, where R – NMDAR; A – agonist of NMDAR (glutamate, for simplicity glycine is not shown); B – blocker (ATL); RA_2 – closed receptor with bound agonist molecules; R^*A_2 -activated receptor with open channel; R^*A_2B – receptor with open, but blocked channel; RA_2B – receptor with bound agonists and the blocker trapped in the ionic pore by channel closure; RB – receptor without agonists with blocker trapped inside the ion pore. Dotted lines show that the blocker can escape closed channels. Agonist bound receptors (RA_2) can undergo reversible calcium-dependent inactivation (RA_2D) (desensitized state). The agonist bound receptor with blocker inside the pore (RA_2B) can also probably undergo calcium-dependent inactivation (RA_2DB).

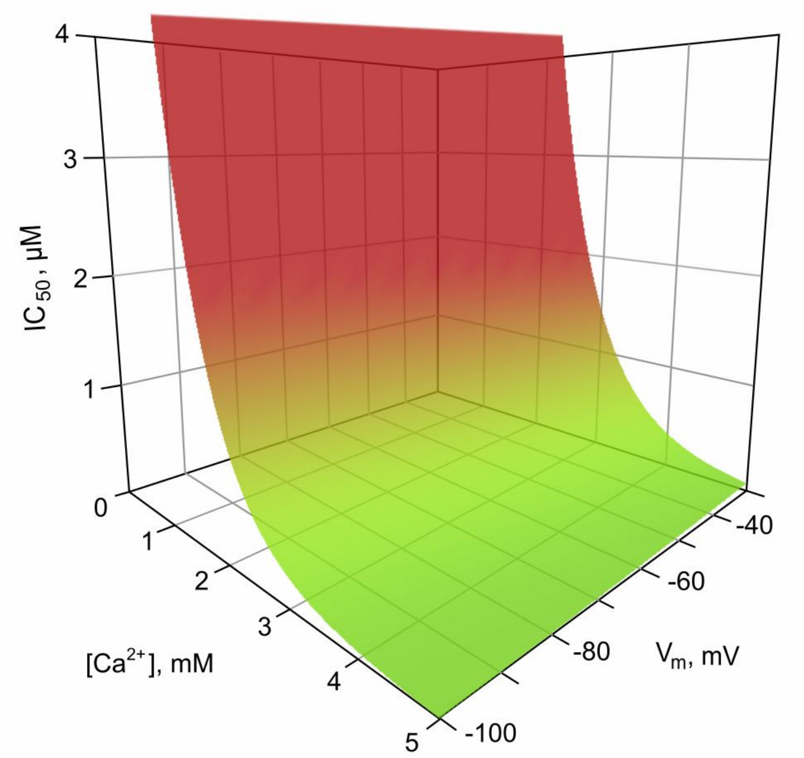

Figure S3. The fragment (below 4 µM ATL) of 3D-plot for $IC_{50}^{Vm,\ [Ca2+]}$ (Fig. 3) presented at high IC_{50} resolution, corresponding to ATL concentrations achieved in the blood serum during therapy indicated (green). The chart exhibits a predominant contribution of Ca^{2+}-dependent inhibition of NMDAR currents by ATL at therapeutic concentrations.
